# Meningioma: A Review of Epidemiology, Pathology, Diagnosis, Treatment, and Future Directions

**DOI:** 10.3390/biomedicines9030319

**Published:** 2021-03-21

**Authors:** Christian Ogasawara, Brandon D. Philbrick, D. Cory Adamson

**Affiliations:** 1 Department of Surgery, University of Hawaii School of Medicine, Honolulu, HI 96813, USA; cogasawa@hawaii.edu; 2 Department of Neurosurgery, Emory University School of Medicine, Atlanta, GA 30322, USA; brandon.philbrick@emory.edu; 3 Department of Neurosurgery, Atlanta VA Medical Center, Atlanta, GA 30322, USA

**Keywords:** meningioma, central nervous system, tumor, benign, malignant

## Abstract

Meningiomas are the most common intracranial tumor, making up more than a third of all primary central nervous system (CNS) tumors. They are mostly benign tumors that can be observed or preferentially treated with gross total resection that provides good outcomes. Meningiomas with complicated histology or in compromising locations has proved to be a challenge in treating and predicting prognostic outcomes. Advances in genomics and molecular characteristics of meningiomas have uncovered potential use for more accurate grading and prediction of prognosis and recurrence. With the study and detection of genomic aberrancies, specific biologic targets are now being trialed for possible management of meningiomas that are not responsive to standard surgery and radiotherapy treatment. This review summarizes current epidemiology, etiology, molecular characteristics, diagnosis, treatments, and current treatment trials.

## 1. Introduction

Meningiomas are the most common primary central nervous system (CNS) tumors [1]. They are usually benign, slow growing neoplasms that are thought to arise from meningothelial (arachnoid) cells (MECs) [1,2,3]. Despite having a reputation of a benign disease, these dural-based tumors can lead to morbidity, presenting with a variety of non-specific, location dependent symptoms. This review discusses the recent 2016 updates to the World Health Organization (WHO) classification of CNS tumors, epidemiology, and etiological/risk factors of meningiomas. This review also covers molecular characteristics and potential applications for grading, clinical features, diagnostics, standard treatment regimens, and ongoing trials of potential treatments.

### 1.1. Meningioma Cell of Origin

MECs are a cellular component of the pia mater, arachnoid mater, and the trabeculae and septae of the subarachnoidal space [4]. They make up a monolayer covering of the meninges and are connected via tight junctions, gap junctions, and desmosomes, providing an interface between neuronal tissue and the cerebrospinal fluid (CSF) [5]. Aside from providing a physical barrier to the CNS and protecting it from mechanical damage, MECs also play a significant role in immunological processes and the maintenance of homeostasis and host defense in the CSF [6,7,8]. Through secretion of pro- and anti-inflammatory chemokines and cytokines, MECs are able to initiate and quench immune reactions [6]. MECs also protect against infection and neurodegeneration via phagocytosis of bacteria and apoptotic bodies, as well as macropinocytosis of neurotoxic peptides and proteins, respectively [6,7,8]. MECs have different embryologic origins depending on their anatomic locations. MECs found at the skull base and cerebral convexity have mesoderm and neural crest origins, respectively [9]. This difference affects the predominating histological subtypes of meningiomas that arise from these cells and the distribution of recurrent somatic mutations [9]. Arachnoid cap cells make up the outer layer of the arachnoid mater and arachnoid villi and with cytological similarities to meningiomas cells, it is likely their cell of origin [10]. Meningiomas are tumors of the meninges but they also occur rarely as primary tumors in the ventricles of the CNS and extracranial organs such as the lungs [3,11], presumably from aberrant MECs.

### 1.2. WHO Grading

The World Health Organization (WHO) grading system for tumors is the standard for grading meningiomas [3]. The most current guidelines (WHO 2016) classified meningiomas into 15 subtypes across 3 grades on the basis of histologic criteria (Table 1) [3]. This grading system correlates with the risk of recurrence and overall survival and, therefore, having major implications on treatment strategy. WHO grade I makes up 80.5% of all meningiomas and has benign histology and indolent behavior [1,3]. WHO grades II and III make up 17.7% and 1.7% of meningiomas, respectively, and have atypical to malignant histology that demonstrates a more aggressive clinical course [1,3,12]. Meningiomas of any subtype with a high proliferation index has a greater likelihood of recurrence and aggressive behavior, and are associated with grade WHO grades II and III meningiomas [3]. Ki-67 proliferation index of >4% and >20% have an increased risk of recurrence and mortality, respectively [3]. Unlike glioma brain neoplasms, the current WHO classification system does not incorporate any genomic or molecular features.

### 1.3. Epidemiology

**Figure 1:**
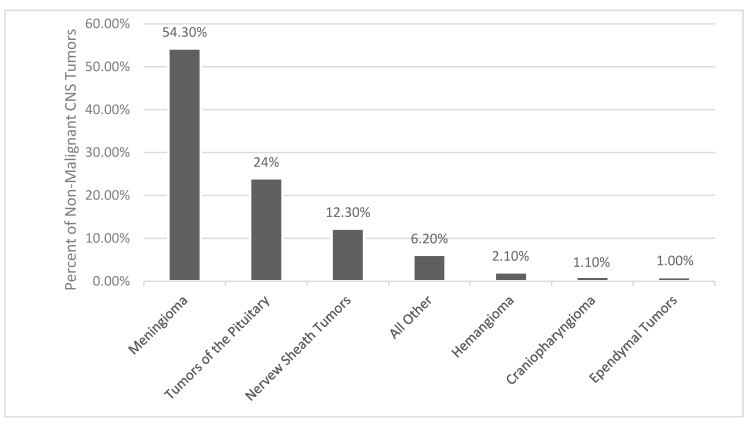
Distribution of non-malignant primary brain and other central nervous system (CNS) Tumors [1].

As reported by histology, meningiomas comprises 37.6% of all primary CNS tumors and 53.3% of all benign CNS tumors (Figure 1) [1]. The incidence of meningiomas increases with age, with the median age at diagnosis being 66 years old [1]. The incidence rate in patients age 40+ years is 18.69/100,000 and in age 0–19 years it is 0.16/100,000 [1]. In patients age 40+ years, age 15–39 years, and age 0–14 years, meningiomas make up 43.6%, 15.6%, and 1.7% of all CNS tumors, respectively [1]. Benign and malignant meningiomas are more common in females, with incidence rate ratios of 2.33 and 1.12, respectively [1]. Females and males in the age 0–19 year range had similar incidence ratios of meningiomas [1]. Children most often have higher grade meningioma with a higher risk of recurrence and decreased overall mortality [13,14]. Benign and malignant meningiomas were also more common in Black people versus Whites people, with incidence ratios of 1.18 and 1.52, respectively [1]. At autopsy, incidental meningiomas were usually found in 2–3% of patients [15,16]

### 1.4. Etiology/Risk Factors

#### 1.4.1. Ionizing Radiation

Currently, ionizing radiation (IR) exposure is the only environmental risk factor identified for meningioma, with reported risks ranging from 6–10 fold increases in incidence [17]. Meningiomas are the most common brain neoplasm caused by IR [18]. Meningioma formation is seen in high and low dose IR [19]. A dose of only 1–2 Gy to the head administered during childhood can lead to a 9.5 fold increase in the incidence of meningiomas, whereas doses of >2.6 Gy are associated with a relative risk of 18.82 for low-grade meningiomas, displaying a positive association with dose increases [19,20,21]. Patients treated with a low dose (<10 Gy), moderate dose (10–20 Gy), and high-dose (>20 Gy) of radiation had latency periods of 35.2 years, 26.1 years, and 19.5 years, respectively, which displays an inverse relationship between dose amount and latency period [19]. Hiroshima and Nagasaki atomic bomb survivors have increased incidence of meningiomas and the risk of developing meningiomas is decreased in patients that were further from the hypocenter [22,23]. Adults treated for tinea capitis as children with scalp irradiation had a significantly higher risk of developing meningioma [24]. Patients with radiation-induced meningiomas (RIM) is frequently present with multiple tumors and have a higher proportion of atypical or anaplastic meningiomas, as well as a higher recurrence rate [19].

#### 1.4.2. Obesity

Positive associations with meningioma risk were identified in body mass index (BMI) and body fat percentage [25]. The summary relative risk (RR) of meningiomas with respect to BMI was 1.48 (95% CI, 1.30–1.69) for obesity and 1.18 (95% CI, 1.07–1.31) for those overweight [26]. In a dose-response analysis, for every 5 kg/m^2^ increment of BMI, the summary RR was 1.19 (95% CI, 1.14–1.25) for meningiomas [26]. Proposed mechanisms for the association of obesity with increased meningioma risk include chronic inflammation and increased adipokine-mediated signaling, as well as insulin signaling and insulin-like growth factor (IGF) signaling [25]. IGF-1 is known to suppress apoptosis and stimulate tumor growth. Higher levels of IGF-1 are observed in both obesity and meningiomas, suggesting a role in the development of these tumors [25]. Unfortunately, there are no clear meningioma-specific molecular pathways associated with obesity.

#### 1.4.3. Occupational (Pesticide/Herbicide)/Diet/Allergies

Increased risk of meningioma was not consistently observed for occupations in the chemical, metal, agricultural, construction, electrical/electronic, and transport sectors [27]. Although it was found that there was a 30% statistically significant increase in the risk of various brain tumors in farmers, a French study did not find an association with pesticide exposure and meningioma risk, even when analysis was restricted to the most exposed subjects [28,29]. Women with occupational herbicide use had a significantly increased risk of developing meningiomas (OR = 2.4, 95%CI: 1.4–4.3), with significant trends of increasing risk with increasing years of herbicide exposure and increasing cumulative exposure [30]. There was no association found between diet and meningiomas [31]. Allergic conditions (asthma and eczema) are protective against developing meningiomas [32]. With IgE, a biomarker of atopic allergy, acting superiorly to any immunoglobulins in targeting over expressed tumor antigens, it is suggested to have a positive role in natural immune surveillance [32]. Patients with meningiomas have lower serum IgE levels than control subjects [33]. Moreover, a hyper-reactive immune system has been shown to be more capable of recognizing and killing cancerous cells. Macrophages, eosinophils, and mast cells armed with IgE could thus be more potent effectors in antitumor immunity [32].

#### 1.4.4. Hormones

With an increased incidence in females and ∼100% of meningiomas having somatostatin receptor 2 (SSTR2), ~88% progesterone receptors, ~40% estrogen receptors, and ~40% androgen receptors, it was thought that hormones played a slight role in tumor growth [17,34,35,36,37]. Although previous data assessing the relationship between meningioma risk and oral contraceptives (OCP), hormone replacement therapy (HRT), and reproductive factors have been inconsistent and inconclusive, two meta-analysis and multiple case-control and cohort studies have shown an increased risk associated with HRT [38,39,40,41,42,43]. The meta-analysis by Qi et al. also showed that there were associations between a postmenopausal state and parity with meningioma development, but no significant associations for OCP use, age at menarche, age at menopause, or age at first birth was observed [38]. Hormone therapy inhibiting estrogen and progesterone receptors has failed to provide clinical benefit [44,45].

### 1.5. Molecular Characteristics

Advances in molecular techniques over the last decade that include genomic and epigenomic data associated with meningiomas have been used to identify genetic biomarkers that may predict tumor behavior and prognosis [46].

#### 1.5.1. Cytogenetics

Chromosomal instability has repeatedly been shown to be one of the most frequent molecular alterations for tumor recurrence and prognosis (Table 2) [12,47,48]. Accumulation of cytogenetic aberrations correlates with increasing tumor grades and aggressiveness, with higher-grade (atypical and anaplastic) meningiomas demonstrating an increasingly complex cytogenetic profile compared to benign meningiomas (6.9 events for high-grade vs. 1.7 events for low-grade) [47,49,50,51,52,53,54]. Sporadic higher-grade meningiomas and lower-grade meningiomas that recur and progress to higher-grade meningiomas both initially demonstrate a higher number of cytogenetic aberrations [47,55,56]. The number of cytogenetic aberrations is also strongly associated with risk of recurrence [47,57]. Copy number alterations are more frequent in meningioma treated with radiation [46]. Gains of chromosome 1q, 9q, 12q, 15q, 17q, and 20q, and losses of 1p, 4p, 6q, 9p 10, 14q, 18q, and 22q have been noted [12,48,54]. Loss of chromosome 22q, where neurofibromatosis type 2 (NF2) gene is located is the most common chromosomal abnormality and is found in up to 80% of meningiomas [50,58]. The frequency of this abnormality increases with tumor grade and occurs in 50% and 75–85% in benign and atypical or anaplastic meningiomas, respectively [12].

Aside from the loss of 22q, grade I meningiomas do not display consistent alterations and are typically stable at the cytogenetic level [52]. Angiomatous meningiomas demonstrate a distinct cytogenetic profile of polysomies of at least one chromosome, but often more, especially in chromosomes 5, 13, and 20 [59,60]. The loss of 1p is the second most common abnormality and is related with tumor progression and a higher recurrence rate seen in higher grade tumors [49,52]. It occurs in 13% to 26% of grade I, in 40% to 76% of grade II, and 70% to 100% of grade III [49]. The genes implicated on this chromosomal arm include TP73, CDKN2C, RAD54, EPB41, GADD45A, and ALPL [12]. The loss of 14q is the third most common abnormality and is predictive of tumor recurrence, tumor progression, and is seen in higher-grade tumors [49,52]. It occurs in up to 31% of grade I, 40% to 57% of grade II, and 55% to 100% of grade III [49]. The genes that are inactivated on 14q are the NDRG family member 2 (NDRG2) and maternally expressed gene 3 (MEG3). These, however, are associated with poor prognosis [61,62].

The combination of 1p and 14q loss is shown to be an independent prognostic factor for the WHO grade and is associated with early recurrence and tumor progression [63]. The loss of chromosome 9p are frequently found in anaplastic meningiomas and are rarely seen in benign or atypical grades, predicting short survival and a worse prognosis [49]. Genes found on 9p are tumor suppressor genes cyclin-dependent kinase inhibitor 2A (CDKN2A), p14ARF, and cyclin-dependent kinase inhibitor 2B (CDKN2B) [49]. Amplification of 17q is also more frequently found in anaplastic meningioma with a frequency of 61% vs. 21% and 14% in grade II and grade I, respectively [49]. Frequent losses of 1p, 14q, and 22q were exhibited more in recurrent and progressive tumors than in de novo higher-grade tumors [12]. Overall, loss of 1p, 6q, 14q, and 18q, and gain of 1q were significantly linked to meningioma recurrence [64]. Clearly, increasing malignant biology correlates with increasing chromosomal and genomic abnormality largely due to bystander events. Additional work on some of these specific mutations may help elucidate the key tumorigenic events.

#### 1.5.2. Familial Syndromes

While the majority of meningiomas occurs sporadically, there are many rare familial syndromes that increase the risk of developing these tumors [65]. While exact molecular mechanisms have yet to be elucidated, these familial syndromes might provide insight behind sporadic meningioma tumorigenesis and implications for management [65].

##### Neurofibromatosis Type 2 (NF2)

NF2 is the most common and well-known familial syndrome associated with meningioma risk [2,65]. NF2 is caused by a germline mutation of the NF2 gene on chromosome 22q12 and is inherited in an autosomal dominant pattern [66]. Over 50% of NF2 patients will develop at least one intracranial meningioma in their lifetime [67]. NF2 meningiomas occurs earlier in life with a mean age of 30 years old and are more likely to have multiple lesions. Further, they are more aggressive than sporadic tumors [12,66]. The associated risk of meningiomas in NF2 corresponds to the type and location of mutations within the gene, with a greater risk associated with truncating mutations than nontruncating mutations and with mutations occurring toward the 5′ end of the gene than the 3′ end of the gene [68]. A larger tumor burden and an earlier onset was also associated with truncating mutation by frameshift or nonsense rather than a nontruncating mutation by missense or splice-site [68].

##### Gorlin Syndrome

Gorlin syndrome, also known as basal cell nevus syndrome or Nevoid basal cell carcinoma syndrome, is an autosomal dominant syndrome associated with an increased risk of meningiomas, with 5% of patients developing tumors [12,65]. Aberrant signaling in the sonic hedgehog (SHH) pathway caused by mutations in PTCH1, PTCH2, and SUFU genes, located at 9q22.32, 1p34.1, and 10q24.32, respectively, with PTCH1 being the most common, increases the risk of meningioma development [65]. Activation of this pathway is responsible for normal neural and tumor development [69]. Inactivation of PTCH1 and SUFU, and activation of SMO, have been implicated in tumorigenesis and maintenance [70]. Patients with SUFU mutations are more likely to develop meningiomas compared to PTCH1 or PTCH2 mutations even with the absence of PTCH mutation, which have been found in families with hereditary multiple meningiomas [70,71].

##### Cowden Syndrome

Cowden syndrome (CS) is an autosomal dominant syndrome that is part of the PTEN hamartoma tumor syndrome (PHTS) [65]. It is caused by germline mutations in phosphatase and tensin homolog (PTEN) on chromosome 10q23.31. The incidence of meningiomas in patients with CS was 8.25% [72]. Proteus syndrome, another syndrome associated with PHTS, has also been shown to increase risk for meningiomas [65]. PTEN protein, through its lipid phosphatase activity, suppresses the PI3K-AKT-mTOR (mammalian target of rapamycin) pathway. Dysfunctional PTEN causes increased cell survival, proliferation, and energy metabolism [73]. It is hypothesized that PTEN mutations are unlikely to be associated with initiation and formation of low-grade meningiomas but may contribute to the progression of higher grade tumors [65].

##### Werner Syndrome

Werner syndrome is an autosomal recessive syndrome and patients with this disease are ~36.2 times the standardized incidence ratio (SIR) (95% CI 17.3, 66.5) more likely to develop a meningioma and are more likely to develop them at a younger age [74]. Werner syndrome is caused by a dysfunctional WRN gene, which is located on chromosome 8p12 and encodes for a protein with DNA helicase activity [65].

##### BAP1 Tumor Predisposition Syndrome

Breast cancer (BRCA)1–associated protein 1 (BAP1) tumor predisposition syndrome (BAP1 TPDS) is a germline mutation of the tumor suppressor BAP1 gene located on chromosome 3p21.1 [75]. BAP1 encodes the BRCA1-associated protein 1 which functions in transcription, chromatin modification, and DNA damage response [65]. Further, 1.7% of patients with BAP1 TPDS develop meningiomas [75]. Germline BAP1-mutant meningiomas arise more commonly than in somatic mutations [75]. Inactivation of BAP1 has been seen in a subset of highly aggressive rhabdoid meningiomas and are associated with very poor clinical outcomes, multiple recurrences, and significantly shortened overall survival [76]. Because BAP1 TPDS is associated with other tumors, patients with a potentially high-grade rhabdoid meningioma should be assessed for a family’s history of cancer and BAP1 status of the tumor [76]. BAP1 mutations co-occurring with NF2, FBXW7, or PBRM1 mutations have worse clinical outcomes than those lacking mutations in these additional genes [76].

##### Familial Syndromes Associated with SMARCB1 and SMARCE1

The switch/sucrose nonfermentable (SWI/SNF) family is an ATP-dependent chromatin remodeling complex that regulates gene expression through nucleosome restructuring and is composed of multiple subunits including SMARCE1 and SMARCB1 [12,77]. Germline mutations of SMARCE1 and SMARCB1 have been seen in several families with familial meningiomatosis [65,77]. SMARCE1 is located on chromosome 17q21.2 and is responsible for inducing apoptosis by stimulating expression of CYLD [65]. Families with heterozygous germline SMARCE1 mutations presented with spinal and intracranial clear cell meningiomas [77]. Males with a symptomatic SMARCE1 mutation developed meningiomas in childhood (age range 2–10 years), while females developed tumors later in adolescence or early adulthood (age range 14–30 years) [77]. SMARCE1 mutations are frequently seen in patients that present with spinal meningiomas with clear cell histology [77]. SMARCB1 is located on chromosome 22q11.23 and in a study examining family members, it was found that cranial meningiomas had a predilection for the falx cerebri [78].

##### Other Familial Syndrome

Other syndromes that have been implicated in the development of meningiomas include Li-Fraumeni, Turcot, Gardener, von Hippel-Lindau, Rubinstein–Taybi syndrome, and multiple endocrine neoplasia type I (MEN1) [65,79,80].

#### 1.5.3. Somatic Mutations

##### NF2

In the 1990s, the NF2 gene was discovered to be a major driver of meningioma development [81]. NF2 is a tumor suppressor gene located on chromosome 22q12 that encodes the Merlin protein (also known as schwannomin) [82]. Merlin is a scaffold protein that belongs to the BAND 4.1 FERM gene family [82,83]. Along with linking plasma membrane receptors to the cortical actin cytoskeleton, it indirectly links transmembrane receptors and intracellular effectors to modulate multiple signaling pathways controlling proliferation, survival, cytoskeletal remodeling, cell-cell adhesion, and cell migration [58,82,83]. Loss of NF2 can activate oncogenic pathways, including Ras/mitogen-activated protein kinase, Notch, phosphoinositide 3-kinase (PI3K)/AKT, Hippo, and mammalian target of rapamycin (mTOR) [58]. Deletion of NF2 can be identified in 50–60% of meningiomas [58]. NF2 alterations are seen in 75% of atypical meningioma [11]. NF2 loss promotes the formation of mesenchymal-like cell phenotypes vs. epithelioid-like ones [37]. Decreased NF2 expression is found in 80% of fibrous and transitional meningiomas and in less than 30% of meningothelial meningiomas [37]. NF2 mutations in low-grade tumors present in the lateral and posterior skull base, while high-grade tumors remain present in parasagittal, falcine, torcula, and intraventricular regions [50,58].

##### Non-NF2 Mutations

Around 40% of sporadic meningiomas lack NF2 mutations and driven by other genetic aberrations [12]. Recent genomic studies of meningiomas have elucidated a rich array of recurrent non NF2 mutations, typically in TNF receptor-associated factor 7 (TRAF7), Kruppel-like factor 4 (KLF4), v-Akt murine thymoma viral oncogene homolog 1 (AKT1), RNA polymerase II subunit A (POLR2A), Telomerase reverse transcriptase (TERT), smoothened/frizzled class receptor (SMO), and Phosphadidylinositol-4,5-bisphosphate 3-kinase catalytic subunit alpha (PIK3CA) (Table 2) [12,50,84,85]. Non-NF2 mutations are frequently identified in grade I tumors and are genomically stable, with the absence of large-scale chromosomal amplifications or deletions [46,84]. Most of these mutations do not coexist with NF2 mutations or monosomy 22 [46,84].

TRAF7 mutations are seen in up to ~25% of meningiomas and in all of the secretory subtypes [86]. They are nearly always benign and located in the medial skull base [50]. They are also exclusive of NF2 and SMO mutations and often cooccur with KLF4, AKT1, or PIK3CA mutations [12,86].

KLF4 mutations are present in ~50% of non NF2 mutated tumors and in up to 9–12% of all meningiomas [11,50,77]. KLF4 mutations are more prevalent in grade I tumors and occur frequently with TRAF7 mutations with nearly all secretory subtypes (97%) presenting with both mutations [77,87]. KLF4 mutations are exclusive of NF2 and AKT1 mutations [50].

AKT1 mutations predominately display meningothelial histology and are mostly seen in grade I meningiomas, occurring in 7–12% of grade I tumors [50]. Sequencing of skull-base meningiomas showed AKT1 mutations in ~30% of patients [11]. AKT1 mutations are rare in grade II and III but have a reduced time in recurrence [12]. More than half of the AKT1 mutations cooccur with TRAF7 mutations but are exclusive of NF2, KLF4, and SMO [12]. AKT1 p.Glu17Lys mutation is also found in Proteus syndrome [12].

POLR2A mutations predominately display meningothelial histology and are almost exclusively found in grade I meningiomas, occurring in 6% of grade I tumors [84]. They are exclusive from other mutations and have a low risk of recurrence, with no POLR2A mutations present in high-grade meningiomas [11,84].

TERT promoter mutations are found in 6.4% of all meningiomas and are associated with higher grade tumors with 1.7%, 5.7%, and 20% of tumors making up grade I, II, and III, respectively [11,12]. TERT promoter mutations are associated with an increased time to progression, with median time to progression being 10.1 months in patients with TERT promoter mutations and 179 months in patients without a TERT promoter mutation [88]. These mutations are associated with meningiomas that progressed from grade I tumors rather than de novo atypical meningiomas, which implicates a high recurrence rate and malignant transformation if identified in grade I tumors [12].

SMO mutations are seen in 3–5% of all meningiomas and display meningothelial histology [77]. SMO mutations are seen in grade I tumors and rarely cooccur with other mutations [12]. They are also associated with larger tumor volume and higher risk of recurrence [12].

PIK3CA mutations are found in 4–7% of meningiomas and often cooccur with TRAF7 mutations but are exclusive of NF2, AKT1, and SMO mutations [12]. PIK3CA mutations are commonly seen in grade I tumors and frequently present with meningothelial or transitional histology [12].

TRAF7 mutations share anatomic locations with KLF4 and AKT1 mutations at the sphenoid wing and midline skull base, and anterior midline skull base, respectively [58]. AKT1 mutations also show localization in the spine and foramen magnum [47,89]. POLR2A mutations are preferentially located at the anterior skull base in the tuberculum sellae [84]. TERT promoter mutation was not found to be associated with a specific location and instead presented evenly with tumors at the skull base and convexities [88]. SMO mutations are localized at the medial anterior skull base, near the midline [50]. PIK3CA mutations arose from the anterior tuberculum sellae, sphenoid wing, and clival regions [58].

#### 1.5.4. Epigenetic Modifications

With variability in predicting meningioma behavior with current WHO grading, epigenetic classifications may be superior in predicting tumor recurrence, progression, and prognosis (Table 3) [48,64,90]. DNA methylation profiles are able to distinguish meningiomas from intracranial tumors that are histologically and radiologically similar [79,90]. DNA methylation profiles are also able to identify intracranial meningiomas from metastatic meningiomas and metastatic lesions from endogenous tissue contamination [91]. Methylation profiles are able to distinguish WHO grade I tumors that experience progression and grade II tumors that remain stable. Further, they can more accurately predict recurrence rates and Kaplan–Meir survival curves than WHO grading [90]. Epigenetic modifications and up/downregulation effects of certain genes are associated with tumorigenesis (HOXA5,6,9,11, IGF2BP1, LMO4, MEG3, PENK, TP73, UPK3A, WNK2), growth/aggressiveness (CCND1, CDK5R1, CTNNB1, ENC1, IGFBP2, IGFBP3, IGF2, NDRG2), progression (ALPL, HIF-3α4, HOXA6,9, IGF2BP1, PENK, TIMP3, TP53, UPK3A, WNK2), higher grade (GSTP1, HOXA5, 6,9,11, MAL2, MEG3, TIMP3, WNK2,), malignant transformation (MAL2, RASSF1A, TP73), recurrence (CTGF, TIMP3), and angiogenesis (THBS1) [12,92,93]. A scoring system consisting of HOXA6, HOXA9, PENK, UPK3A, and IGF2BP1 significantly correlated with a high frequency of recurrence [94]. The loss of trimethylation of lysine 27 of histone H3 (H3K27me3) and an overexpression of the histone cluster H1 family member C (HIST1HIc) gene has been implicated in tumorigenesis and progression. Further, it is more frequently found in recurrent meningiomas [12]. Mutations affecting epigenetic modifiers KDM5C and KDM6A (histone demethylases), as well as SMARCB1, were present in 8% of meningiomas [95]. MicroRNA (miRNA) inhibits translation of mRNA into proteins and are implicated in tumorigenesis (miR-21, miR-200a), progression (mir-21, miR-190a, miR-224, miR-335), recurrence (miR-29c-3p, miR-219-5p, miR-190a), and higher grade (miR-145) [12].

Despite the increasing number of studies elucidating the utility of genetic profiles, advanced molecular diagnostics have not yet been integrated in the workup or classification of meningiomas, as has been the case for gliomas [79].

## 2. Clinical Features

### 2.1. Presenting Locations

Meningiomas are most commonly seen in the following areas: convexity (lateral hemisphere) (20–37%); parasagittal (medial area of hemispheres) (13–22%) (includes falcine meningiomas (5%)); spinal (7–12%); skull base (43–51%); frontobasal (10–20%); sphenoid and middle cranial fossa (9–36%); posterior fossa (6–15%); tentorium cerebelli (2–4%); cerebellar convexity (5%); cerebellopontine angle (2–11%); foramen magnum (3%); and petroclival (<1–9%); intraventricular (1–5%); orbital (<1–2%); and ectopic locations (<1%) (Table 4) [2,96,97].

Grade I meningiomas are more likely to be found at the skull base, whereas higher grade meningiomas are more likely to be found at the convexity, parasagittal, falcine, torcular, and intraventricular regions [56,77]. Multiple meningiomas with and without NF2 alterations are present in 1% and 4% of patients, respectively [77]. Because of these characteristic locations for meningiomas, imaging can often be sufficient in the diagnosis.

### 2.2. Signs and Symptoms

The presentation of meningiomas are often non-specific, but location and compression of adjacent brain and vascular structures can lead to focal neurologic deficits (including cranial nerve deficits) [66]. Symptoms that are commonly seen are as follows: headache (33.3–36.7%), focal cranial nerve deficit (28.8–31.3%), seizure (16.9–24.6%), cognitive change (14.4%), weakness (11.1%), vertigo/dizziness (9.8%), ataxia/gait change (6.3%), pain/sensory change (5.6%), proptosis (2.1%), syncope (1.0%), and asymptomatic (9.4%) [96,98].

Skull base meningiomas present more often with neurological deficits and non-skull base meningiomas are more likely to present with seizures [99,100]. Anterior cranial fossa meningiomas (anterior falcine, olfactory groove, or orbitofrontal) are often large at presentation and present with impaired vision (54%), headache (48%), anosmia (40%), seizure (20%), psychomotor symptoms, and behavioral disturbance with personality disintegration [101,102]. Along with gradual personality changes with apathy and dementia, anterior falx meningiomas often present with a long history of headache and optic atrophy [102]. Parasagittal meningiomas can grow to considerable size before presenting with symptoms [102]. They mostly present with Jacksonian seizures of the lower limbs or headache and advanced anterior parasagittal meningiomas, which are characteristically present with papilledema and homonymous hemianopia [102]. Tuberculum sellae meningiomas usually present with insidious unilateral visual loss, followed by scotomatous defects in the other eye [102].

**Figure 2:**
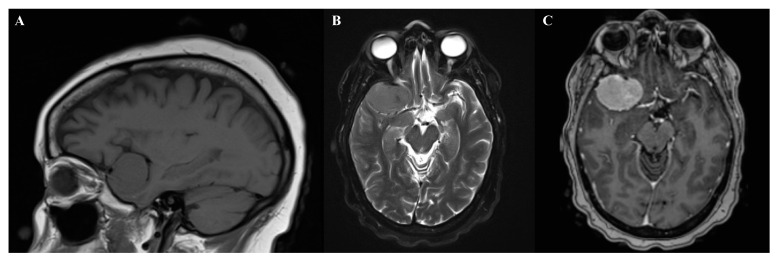
Magnetic resonance imaging of right sphenoid wing meningioma. (**A**) Sagittal T1. (**B**) Axial T2. (**C**) T1+gadolinium contrast.

Suprasellar meningiomas may present with only minor hormonal abnormalities [102]. Lateral sphenoid wing meningiomas often present with painless unilateral exophthalmos, followed by unilateral loss of vision (Figure 2) [102]. Temporal lobe meningiomas frequently presented with seizures [102]. Petroclival meningiomas can present with ataxia and cranial nerve neuropathies such as trigeminal nerve impairment [101,103]. Clinoidal meningiomas often present with a wide variety of visual impairment, cranial nerve palsies, and exophthalmos [102].

Posterior cranial fossa meningiomas can develop obstructive hydrocephalus and present with papilledema and early-morning headache [101]. Peritorcular meningiomas symptoms are commonly caused by compression of the occipital lobe or the cerebellum and present with a headache with occipital localized pain, papilledema, and homonymous field deficits, as well as ataxia, dysmetria, hypotonia, and nystagmus [102]. Spinal meningiomas, which are most common in the thoracic spine, present with slowly progressive spastic paresis with or without radicular or nocturnal pain [101]. Cervical spine and craniocervical junction are the second most common sites of spinal meningiomas and present with spastic quadriparesis with or without low bulbar signs [101]. Meningiomas close to the bone can cause focal hyperostosis and is almost invariably a sign of bone invasion by meningioma cells and can cause bulging of bones and localized pain [102].

Spontaneous bleeding rarely occurs and are seen more in patients less than 30 and older than 70 years old [102]. Spontaneous bleeding has an overall mortality rate of 21% and patients with spontaneous bleeding who are unable to regain consciousness before surgical repair has an overall mortality rate of 75% [104]. Elderly patients 70+ years old are more likely to present with sensory-motor deficits (38.3%) and cognitive impairments (28.8%) [98].

### 2.3. Diagnostics

The initial tentative diagnosis of meningiomas can be made via magnetic resonance imaging (MRI)MRI or contrast-enhanced computed tomography (CT) in patients with contraindications to MRI (e.g., pacemaker) [105]. With high expression of somatostatin receptor 2 (SSTR2) on meningioma cells, positron emission tomography (PET) imaging using SSTR ligands such as 68Ga-DOTATOC and 68Ga DOTATATE has been used as a diagnostic tool and to help delineate healthy tissue from meningiomas [106]. Meningiomas on MRI are usually hypo- to isointense relative to the cerebral cortex on T1-weighted sequences and iso- to hyperintense on T2-weighted sequences, displaying strong homogeneous enhancement following administration of gadolinium contrast [107]. Although not specific and demonstrated in other dural neoplasms, a dural tail can be seen in 72% of meningiomas on postcontrast imaging and can help differentiate meningiomas from other extra-axial tumors such as schwannomas and pituitary adenomas [107]. Heterogenous appearance can be caused by the presence of intratumoral cysts, hemorrhage, or necrosis and may be associated with more aggressive behavior of the tumor [107]. Meningiomas on CT usually appear isodense relative to cerebral cortex but can occasionally be hyperdense or slightly hypodense [105]. Meningiomas usually present as a sharply circumscribed lobular mass with a broad-based dural attachment and demonstrate homogenous enhancement following iodine contrast administration [107]. CT is more sensitive than MRI in detecting hyperostosis, intratumoral calcifications, and interosseous tumor growth [106]

Histological verification helps rule out other diagnosis such as metastasis [106]. Often inconspicuous or absent, meningiomas can present with histologic features such as pathognomonic whorls, and intranuclear cytoplasmic pseudoinclusions and psammoma bodies [79]. Many other dural masses including primary neoplastic processes, metastases, granulomatous diseases and infections can mimic meningiomas [108]. Meningioma mimics presented in the convexity (40%), parafalcine (24%), and skull base (24%). The most common meningioma mimics were hemangiopericytoma/solitary fibrous tumor (HPC/SFT), followed by metastatic lesions and schwannomas [109].

## 3. Treatment

**Figure 3:**
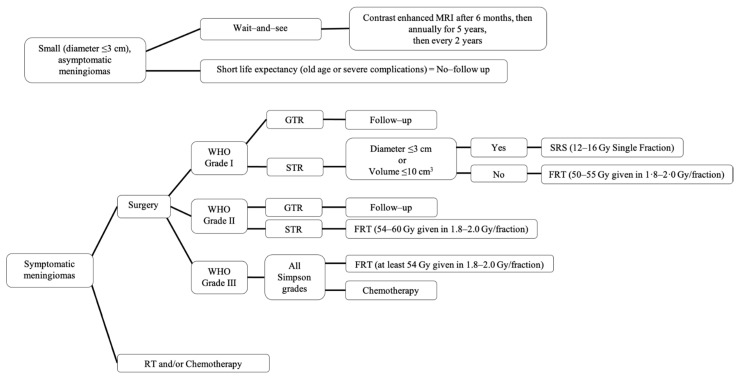
Summary of recommended management of meningiomas. SRS, stereotactic radiosurgery; FRT, fractionated radiotherapy; GTR, gross total resection; STR, subtotal resection.

Treatment for meningiomas is highly individualized and includes a combination of observation, surgical resection, radiotherapy, and rarely chemotherapy (Figure 3) [106]. The potential consequences of different treatments can vary greatly [106]. Through recent advances in advancements in neurosurgery, neuroimaging, and neuroanesthesia, patients are experiencing better long-term outcomes, retreatment free survival, and overall survival [110].

The “wait-and-see” observation approach is a common strategy used for patients with incidentally diagnosed meningiomas that are small (tumor diameter ≤3 cm) and asymptomatic [111,112]. These patients are observed and followed with MRI scans until they become symptomatic or until their tumors are considered large enough to treat [111] in order to prevent future symptoms. Some tumors will not progress. According to the European Association of Neuro-Oncology (EANO), after initial diagnosis they suggest performing a contrast enhanced MRI 6 months later to evaluate for tumor changes [106]. If the patient remains asymptomatic, they can be followed up annually for 5 years and then every 2 years thereafter [106]. Patients that have a clear radiological diagnosis of benign meningioma with shorter life expectancy due to old age or severe complications do not need to be observed [106].

Surgical resection is the primary choice for symptomatic, observation failure meningiomas, or large tumors that are anticipated to causes symptoms soon. GTR can cure the majority (70–80%) of patients [11,102,112]. The goal for surgery is GTR (Simpson I, GTR); however, the ability to achieve this may be limited by various factors, including tumor location, involvement of venous sinuses and neurovascular tissue, and other patient factors affecting safety of surgery in general [79]. These factors influence the decision to pursue surgery, the surgical approach, and the extent of resection [79]. The extent of resection, defined by the Simpson grade (Table 5), heavily impacts the rates of recurrence for surgically treated meningioma of all WHO grades [106]. The Simpson grade is defined by postoperative imaging and the neurosurgeon’s assessment [79]. Over time, there has been an increase in the rate of GTR achieved [110]; however, most neurosurgeons focus on a better functional outcome over tumor resection outcome.

Radiation therapy (RT) has become a first-line treatment for unresectable meningiomas, such as certain skull base meningiomas that have encased neurovascular structures [112]. With a lack of data from randomized controlled clinical trials comparing different RT for meningiomas, most RT data is based on retrospective studies [79]. In WHO grade I, meningiomas after subtotal resection (Simpson IV, STR) or in the setting of recurrence of previously resected meningiomas, stereotactic radiosurgery (SRS), or fractionated radiotherapy (FRT) can be offered [106]. SRS (12–16 Gy single fraction) is used in small tumors (<3 cm in diameter or 10 cm^3^ in volume) and FRT (50–55 Gy given in 1.8–2.0 Gy per fraction) is used when the tumor volume cannot be treated with a single fraction [106]. Even after Simpson I resection, WHO grades II and III meningiomas have a high risk of recurrence (30–40% and 50–80% after 5 years, respectively) [112]. Therefore, adjuvant RT is often apart of initial treatment after surgery in WHO grades II and III meningiomas, with FRT being preferred over SRS [112]. Data for RT in WHO grade II meningiomas after GTR remains unclear but it is recommended that WHO grade II STR receive adjuvant FRT (54–60 Gy given in 1.8–2.0 Gy per fraction) and WHO grade III receive adjuvant FRT (at least 54 Gy given in 1.8–2.0 Gy per fraction), regardless of GTR or STR [106,112].

Patients who develop recurrent or progressive meningiomas that no longer respond to surgery or radiotherapy are treated with salvage systemic therapy [112]. The EANO considers the use of systemic therapy to be experimental with only level C evidence, thus no specific recommendations are given [106]. The National Comprehensive Cancer Network (NCCN) recommends the use of α-IFN, somatostatin receptor agonists, and vascular endothelial growth factor (VEGF) inhibitors for the treatment of meningioma [113]; however, efficacy is greatly limited.

## 4. Outcomes and Natural History of Meningioma

Meningiomas are typically slow growing, with a linear growth rate of 2–4 mm/year for asymptomatic meningiomas [79]. However, a third of all meningiomas experience no growth and of the meningiomas that grow, 25% experience exponential growth [2]. The most reliable prognostic factors of meningiomas are the histological grade (WHO grade) and the extent of tumor resection (Simpson grade) [12]. The 10-year overall survival rate of WHO grades I, II, and III tumors are 83.7%, 53%, and 0%, respectively, despite aggressive therapy efforts [1,79]. Benign and malignant spinal meningiomas had a higher 10 year survival rate of 95.6% and 73.4%, respectively, than benign and malignant cerebral meningiomas of 83.2% and 55.7%, respectively [1].

The 5-year recurrence rates of WHO grades I, II, and III tumors after Simpson grade I GTR are 7–23%, 50–55%, and 72–78%, respectively [2]. After 15 years, almost all STR patients relapse, 60% of which died, and most occurred within 10 years [112].

Metastasis is an exceedingly rare complication, estimated to occur in ~0.1% of meningiomas and most of which are WHO grade III [3]. The most common sites of metastasis are lung (60%) and pleura, followed by the bone, liver, lymph nodes, and kidneys [51,114]. In rare cases, WHO grade I meningiomas may metastasize to the lung, though the prognosis is surprisingly good [51]. Of the patients that received surgery, 12.3% developed new postoperative seizures and 40% developed cognitive or emotional problems (e.g., anxiety or depressive symptoms) [100,115].

## 5. Current Areas of Research

Radiotherapy remains the most widely used and studied adjuvant therapy for meningiomas; however, many questions remain. Most agree that there is no role for RT for WHO grade I tumors, unless in the setting of unresectable symptomatic initial or recurrent tumors. While the role of radiotherapy in WHO grade II GTR tumors not fully elucidated, multiple phase II and randomized controlled trials are trying to shed light on this issue [116]. A phase II trial (RTOG 0539) demonstrated that their intermediate risk group (newly diagnosed WHO grade II GTR (69.2%) and recurrent WHO grade I with any resection extent (30.8%)), when treated with RT (standard dose of 54 Gy), had a 98.3% 3-year progression-free survival (PFS) and 96% 3-year overall survival (OS) (Table 6) [117]. Another phase II trial (EORTC 22042-26042) demonstrated that WHO grade II GTR meningiomas treated with adjuvant RT using a high-dose of 60 Gy had a 88.7% 3-year PFS and 98.2% 3-year OS [118]. Currently, randomized controlled trials ns20191111 (NCT04127760) and NRG-BN003 (NCT03180268) are looking at 3-, 5-, and 10-year OS and PFS in grade II GTR meningiomas that receive adjuvant RT, and the ROAM/EORTC-1308 trial comparing at least 5-year OS and PFS [119].

**Figure 4:**
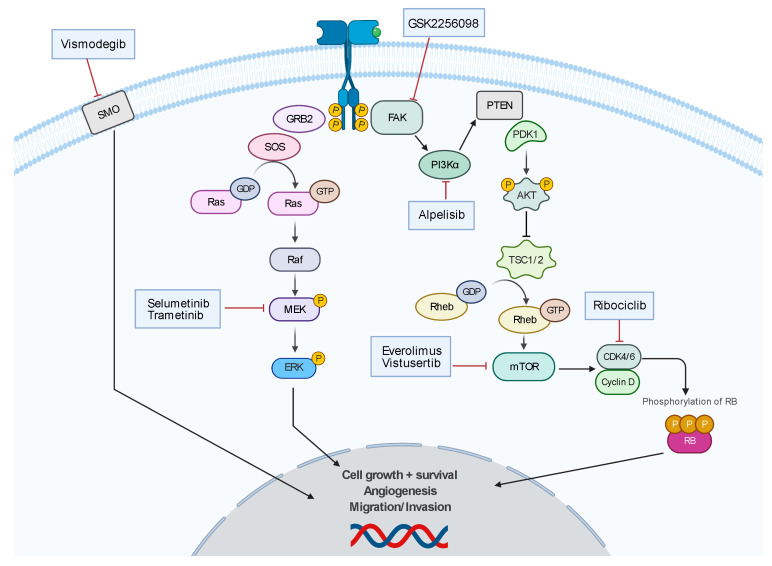
Summary of developing chemotherapy treatments.

There are no established chemotherapies for meningioma; however, there is a robust research effort (Figure 4). There are ongoing clinical trials investigating chemotherapeutics that target molecular mutations such as vismodegib and GSK2256098 (NCT02523014), which are inhibitors of SMO and FAK, respectively. There are other clinical trials investigating pathway-directed therapies such as MEK pathway inhibitor, selumetinib (SEL-TH-1601, NCT03095248), CDK-p16-Rb pathway inhibitor, ribociclib (LEE-011, NCT02933736), and mTOR-pathway inhibitor, everolimus (NCT01880749 and NCT01419639), and vistusertib (AZD2014, NCT03071874). The ALTREM clinical trial is investigating the co-administration of phosphoinositide 3-kinase α (PI3Kα) specific inhibitor, alpelisib, and the MEK inhibitor, trametinib (NCT03631953). The phase II CEVOREM trial demonstrated that the coadministration of everolimus and octreotide (SSTR2A agonist) had a 6-month PFS of 55%, and 6- and 12-month OS of 90% and 75%, respectively [120]. The CEVOREM trial showed more than a 50% decrease in the growth rate at 3 months in 78% of tumors and the median tumor growth rate over 3 months decreased from 16.6% before treatment to 0.02% at 3 months and 0.48% at 6 months after treatment [120]. The NCT02831257 trial demonstrated that patients treated with AZD2014 had a 6-month PFS of 88.9% and 5.6% (1/18) of patients experienced a decrease in tumor volume of at least 20% compared to baseline.

**Figure 5:**
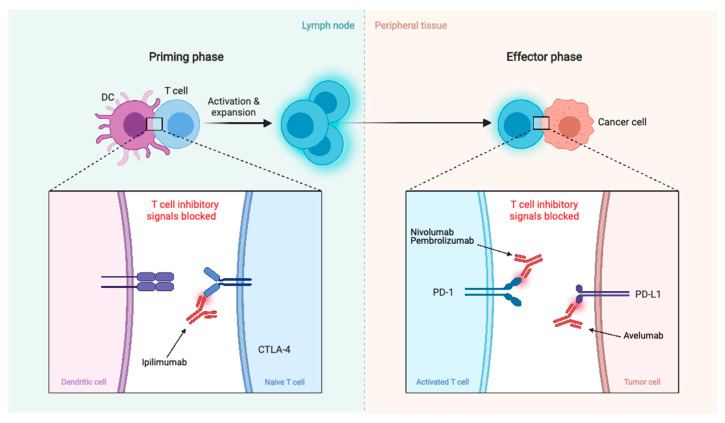
Summary of immunotherapy treatments. Blockage of CTLA-4, PD-1, and PD-L1 promotes an effective immune response against cancer cells.

There are also clinical trials investigating immunotherapy agents such as checkpoint inhibitors PD-1 antagonist, nivolumab (NCT02648997, NCT03173950, and NCT03604978 in combination with ipilimumab), another PD-1 antagonist, pembrolizumab (NCT03279692, NCT03016091, and NCT04659811 in combination with stereotactic radiosurgery), and PD-L1 antagonist, avelumab (NCT03267836 in combination with proton radiotherapy) (Figure 5).

## 6. Conclusions

Meningiomas are mostly benign tumors originating from MECs, which make up 37.6% of all primary CNS tumors. Meningiomas are more common in females and the incidence rate increases with age. Ionizing radiation and specific molecular alterations have been associated with meningioma development. Meningiomas are classified into 15 subtypes across 3 grades with survival and recurrence rates worsening as their grade increases. Initial diagnosis is based on MRI or contrast-enhanced CT. For tumors that are small and asymptomatic, a wait-and-see approach is taken, while complete surgical excision is the optimal treatment for symptomatic meningiomas. Radiotherapy is used in often used for symptomatic primary or recurrent grade I meningioma. We most strongly recommend postoperative radiotherapy for grade II GTR/STR and grade III anaplastic/malignant GTR/STR. While systemic therapy is still under investigation, it is reserved for meningiomas that are recurrent or progressive that no longer respond to surgery and radiotherapy.

With current imaging and histopathologic grading suffering from subjectivity and variability in diagnostic and prognostic power, the incorporation of genomic and molecular features may provide a better system for classification. An integrated diagnostic protocol can improve the accuracy in predicting recurrence and outcome, and can help tailor specific treatment plans for individual patients. Although key mutations and signaling pathways are being uncovered, there is still a lack of targeted systemic therapies, though there are many clinical trials underway.

## Figures and Tables

**Table 1:** Overview of 2016 World Health Organization (WHO) classification system for grading meningiomas. HPF, high-power fields; N/C, nuclear/cytoplasmic.

	Grade I	Grade II	Grade III
Histologic Subtypes	Meningothelial Fibrous Transitional Psammomatous Angiomatous Microcystic Secretory Lymphoplasmacyte-rich Metaplastic	Atypical Clear cell Choroid	Anaplastic Rhabdoid Papillary
Diagnostic Criteria	-Presence of <4 mitoses per 10 HPF	-Presence of 4–19 mitoses per 10 HPF or -Brain invasion or -At least 3/5 of the following: -Patternless sheeting architecture -Small cell formation with high N/C ratio -Prominent nucleoli -Hypercellularity -Spontaneous intratumoral micronecrosis	-Presence of ≥20 mitoses per 10 HPF or -Overtly malignant morphology (carcinomatous, sarcomatous, and melanomatous cytology)

**Table 2:** Somatic and chromosomal mutations in relation to the WHO grade and subtype variants.

	Grade I	Grade II	Grade III
Subtype (WHO grade)	Meningothelial *(TRAF7, AKT1, SMO,* *PIK3CA, POLR2A)* Fibrous *(NF2)* Transitional *(NF2, PIK3CA, AKT1)* Psammomatous *(NF2)* Angiomatous Microcystic Secretory *(KLF4, TRAF7)* Lymphoplasmacyte-rich Metaplastic	Atypical *(NF2, TRAF7, AKT1, TERT)* Clear cell *(SMARCE1)* Choroid	Anaplastic *(NF2, TERT)* Rhadoid *(BAP1)* Papillary
Gene Mutation	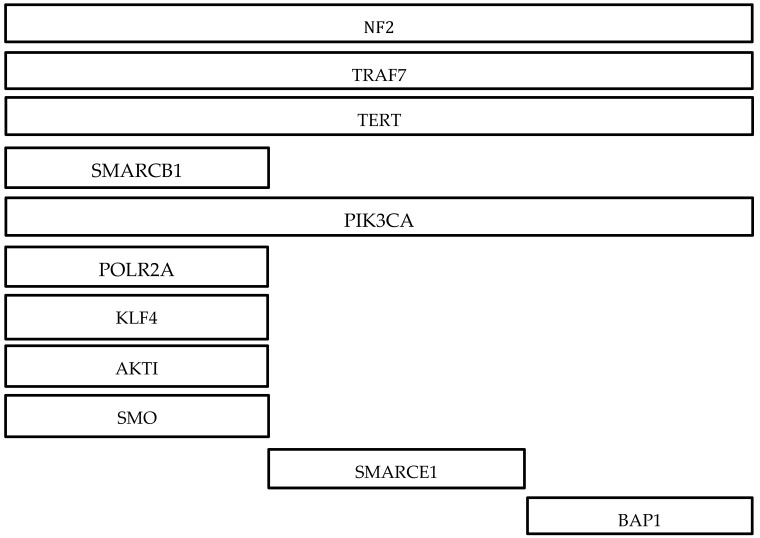		
Chromosomal Alterations	Loss: 22q 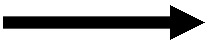	Loss: 1p, 6q, 10, 14q, 18q Gain: 1q, 9q, 12q, 15q, 17q, 20q 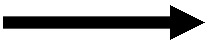	Loss: 9p Amplification: 17q

**Table 3:** Epigenetic modifications in meningiomas.

Affected Genes	Possible Role in Meningioma
Methylation/up/down regulation	
*ALPL*	Progression
*CCND1*	Growth and aggressiveness
*CDK5R1*	Growth and aggressiveness
*CTGF*	Recurrence
*CTNNB1*	Growth and aggressiveness
*ENC1*	Growth and aggressiveness
*GSTP1*	Higher grade
*HIF-3α4*	Progression
*HOXA5*	Tumorigenesis, higher grade
*HOXA6*	Tumorigenesis, progression, higher grade
*HOXA9*	Tumorigenesis, progression, higher grade
*HOXA11*	Tumorigenesis, higher grade
*IGFBP2*	Growth and aggressiveness, progression
*IGFBP3*	Growth and aggressiveness
*IGF2*	Growth and aggressiveness
*IGF2BP1*	Tumorigenesis
*LMO4*	Tumorigenesis
*MAL2*	Higher grade, malignant transformation
*MEG3*	Tumorigenesis, higher grade
*NDRG2*	Growth and aggressiveness
*PENK*	Tumorigenesis, progression
*RASSF1A*	Malignant transformation
*THBS1*	Angiogenesis
*TIMP3*	Progression, higher grade, recurrence
*TP53*	Progression
*TP73*	Tumorigenesis, malignant transformation
*UPK3A*	Tumorigenesis, progression
*WNK2*	Tumorigenesis, progression, higher grade
Histone Modifications	
*HIST1HIc*	Tumorigenesis, progression
H3K27me3	Recurrence
KDM5C	Grade 1, III
KDM6A	Grade II
Chromatin Remodeler	
*SMARCB1*	Higher grade
*SMARCE1*	Higher grade
microRNA	
miR-21	Tumorigenesis, progression
miR-29c-3p	Recurrence
miR-145	Higher grade
miR-190a	Progression, recurrence
miR-200a	Tumorigenesis
miR-219-5p	Recurrence
miR-224	Progression
miR-335	Progression

**Table 4:** Meningioma locations with associated mutations.

Location	Frequency (%)
Convexity	20–37%
Parasagittal (*NF2*)	13–22%
Falcine (*NF2*)	5%
Spine (*AKT1*)	7–12%
Skull Base	43–51%
Frontobasal (*TRAF7, AKT1, POLR2A, PIK3CA, SMO*)	10–20%
Sphenoid and Middle Cranial Fossa (*TRAF7, AKT1, PIK3CA*)	9–36%
Posterior Fossa (*NF2*)	6–15%
○ Tentorium Cerebelli	2–4%
○ Cerebellar Convexity	5%
○ Cerebellopontine Angle	2–11%
○ Foramen Magnum (*AKT1*)	3%
○ Petroclival (*PIK3CA*)	<1–9%
Intraventricular (*NF2*)	1–5%
Orbital	<1–2%
Ectopic locations	<1%

**Table 5:** Simpson grades of meningioma resection.

Extent of Resection	Simpson Grade	Description
Gross Total Resection (GTR)	Grade 1	Gross total resection of tumor, dural attachment, and involved bone (extradural extension)
	Grade 2	Gross total resection of tumor, coagulation of dural attachment
	Grade 3	Gross total resection of tumor without resection of coagulation of dural and extradural components
Subtotal Resection (STR)	Grade 4	Partial (subtotal) resection of tumor
--	Grade 5	Biopsy only

**Table 6:** Summary of current areas of research.

Type of Therapy	Study/Agent	Dose/Target	Results	Trial ID
Radiotherapy	WHO grade II GTR meningiomas w/adjuvant RT	54 Gy in 1.8 Gy per fraction	--	NCT04127760
		59.4 Gy in 33 fractions of 1.8 Gy each	--	NCT03180268
		60 Gy in 30 fractions	--	ISRCTN71502099
		54 Gy in 30 fractions of 1.8 Gy each	3-year PFS = 98.3% 3-year OS = 96%	NCT00895622 (RTOG 0539)
		60 Gy in 30 fractions	3-year PFS = 88.7% 3-year OS = 98.2%	NCT00626730 (EORTC 22042-26042)
Chemotherapy	Vismodegib GSK2256098	SMO FAK	--	NCT02523014
	Selumetinib	MEK pathway	--	NCT03095248
	Ribociclib	CDK-p16-Rb pathway	--	NCT02933736
	Everolimus	mTOR-pathway	--	NCT01880749 NCT01419639
	Everolimus + Octreotide	mTOR + SSTR2A	6-month PFS = 55% 6-month OS = 90% 12-month OS = 75%	NCT02333565 (CEVOREM)
	Vistusertib (AZD2014)	mTOR-pathway	--	NCT03071874
			6-month PFS = 88.9% Decrease in tumor volume of at least 20% = 5.6%	NCT02831257
	Alpelisib Trametinib	Pi3Kα inhibitor MEK inhibitor	--	NCT03631953
Immunotherapy	Nivolumab	PD-1	--	NCT02648997 NCT03173950
	Nivolumab w/Multi-Fraction SRS ± Ipilimumab	PD-1 ± CTLA-4	--	NCT03604978
	Pembrolizumab	PD-1	--	NCT03279692 NCT03016091
	Pembrolizumab w/SRS	PD-1	--	NCT04659811
	Avelumab w/Proton radiotherapy	PD-L1	--	NCT03267836

## Data Availability

Not applicable.
